# Advancing the Role of Food Processing for Improved Integration in Sustainable Food Chains

**DOI:** 10.3389/fnut.2020.00034

**Published:** 2020-04-03

**Authors:** Dietrich Knorr, Mary Ann Augustin, Brijesh Tiwari

**Affiliations:** ^1^Food Biotechnology and Food Process Engineering, Technische Universität Berlin, Berlin, Germany; ^2^CSIRO Agriculture and Food, Werribee, VIC, Australia; ^3^Teagasc Food Res Ctr, Food Chem & Technol, Dublin, Ireland

**Keywords:** sustainability, food processing, emerging technologies, new raw materials, food chain

## Abstract

Food scientists need to work together with agriculturists, nutritionists, civil society, and governments to develop an integrative approach to feed a growing population sustainably. Current attention on food sustainability mainly concentrates on production agriculture and on nutrition, health, and well-being. Food processing, the necessary conversion of raw materials to edible, functional, and culturally acceptable food products, is an important link between production and consumption within the food value chain. Without increased attention to the role of food processing for a maintainable food supply, we are unlikely to succeed in addressing the mounting challenges in delivering sustainable diets for all people. The objective is to draw on multidisciplinary insights to demonstrate why food processing is integral to a future food supply. We aim to exemplify the importance of essential relevant sustainability indicators and impact assessment for developing informed strategies to feed the world within planetary boundaries. We provide a brief outlook on sustainable food sources, review food processing, and recommend future directions. We highlight the challenges and suggest strategies for improving the sustainability of food systems, to hopefully provide a catalyst for considering implementable initiatives for improving food and nutrition security.

## Introduction

A sustainable future food supply in the face of depleting natural resources, climate change, rapid urbanization, changing demographics, and a growing global population is a global challenge. The demand for resources has paralleled the history and development of mankind. The period of unprecedented growth and the scientific and technological progress after the two World Wars in the twentieth century caused significant depletion to existing resources and the environment. During that period, the term “sustainability,” or the more appropriate term in the German language *Nachhaltigkeit*, meaning “lastingness,” emerged (1). The likely most influential reports during that period were “Fritz” Schumacher's “Small Is Beautiful” (2), which called for a return human measure and intermediate technologies and the report of the Club of Rome on “The Limits of Growth” (3).

Increasingly, the focus is on providing sustainable diets that have low environmental impacts and improving the well-being of populations both now and into the future (4). There is an imperative for all parts of the food chain, from production, processing, packaging, storage, to the delivery of food to the consumer, to take steps to make efficient use of resources in each of their operations to ensure healthy diets for an increasing population, against changing demographics, and an increasingly urbanized world (5).

Food processing is a critical element in the food supply chain. Processed foods have been part of civilization since ancient times. Fire use by humans is related to cooking. Knapped stones in Kenya, dating back to 1.5 million years ago, were identified as being evidence of exposure by humans to high heat (6). Meat has been roasted more than 1.8 million years; bread made 30,000 years ago; beer, wine, and cheese first produced between 7,000 and 5,000 bc, and olive oil and palm oil introduced between 5,400 and 3,000 bc. Other foods available in the bc era include pickles, noodles, chocolate, bacon, fermented flavorings, and sugar, and with many other foods introduced through the ages (7). Even in ancient times, both primary (e.g., drying, milling, oil extraction) and secondary processing (i.e., when products of primary processing are formulated and manufactured into processed foods) were employed to convert produce into safe and palatable foods and to extend shelf life. There have been major brands and processing companies that capitalized on the demand for processed food for the masses through the years. Food processing also creates important opportunities for generating income and employment for communities (8). Processed foods are an integral part of today's diet and a significant contributor to food and nutrition security (9).

## Sustainable Food Systems

Sustainable food systems are fundamental to ensuring sustainable development and securing food and nutritional security for all populations. The first book dealing with sustainable food systems (10) states in its preface that “…efforts have remained limited in scope, and they often provide only sporadic responses to far-reaching problems.” Unfortunately, even today these statements may hold true. Pimentel and Pimentel (11) compared fossil energy requirements vs. US daily food energy intake for various diets and demonstrated that an ~10-fold fossil energy is needed to produce daily food energy intake for non-vegetarian diets compared to vegetarian diets. A US National Academy of Science—National Research Council selection of unsolved food science—related problems and suggested approaches for solutions in developing countries from 1974 (12) reads like a fairly recent document, suggesting the lack of progress in the area.

The current European Strategy and Policy Analysis System report (13) points out that food and water supply will be about managing scarcity. The Food and Agriculture Organization (FAO) High Level Panel of Experts report on Food Security and Nutrition (14) recommends that the key points of intervention to manage scarce resources is through maximizing nutrition entering and minimizing nutrition exiting the food supply chain. Another HLPE report provides insights into agroecological and other innovative approaches for sustainable agriculture and food systems that enhance food security and nutrition (15). Willett et al. (16) suggested sustainability should be central to food security, as the focus previously has been mainly on food production and nutrition issues, and there was an omission of the critical part of food processing/preparation within the food chain. Lillford and Hermansson (17) stressed these omissions by addressing key global challenges and the critical needs of food science and technology including the design of sustainable processes and engineering systems, and the use of emerging processes requiring reduced water and energy as one of the key missions for food science and technology.

For the sustainability of the food processing industry, the challenge is to develop strategies that improve social, environmental, and economic sustainability (18), while staying within planetary boundaries, addressing the United Nations' sustainable development goals and The Natural Step performance targets for sustainability, so that the food industry maintains its social license to operate (19). Of the 17 sustainable development goals of the United Nations, 10 have been related to food sustainability. Food demand makes up 26% of the global ecological footprint. The Earth Overshoot Day 2019, the day all humanity has used the nature's resource budget for the entire year, was July 29. This date is the earliest ever and that even at the current rate we would need 1.75 planet earths rather than the one we have, or as the French philosopher and sociologist Latour (20) puts it, “We have come down to Earth.”

## Sustainability Indicators and Impact Assessment

Bettencourt and Kaur (21) stated “The concepts of sustainable development have experienced extraordinary success since their advent in the 1980s…. However, it remains unclear how far the field has progressed as a scientific discipline.” There is a lack of internationally accepted standards for where, when, and what indicator to employ for measuring sustainability. Various indicators have been used to measure sustainability, but most cover environmental, social, and economic aspects, with additional specific indicators and finer degrees of granularity used by different authors.

Early work on sustainable benchmarking of supply chains included pollution, labor standards, and ethics in food supplier relations and waste issues, as well as environmental, social, and economic measures. The “Brundtland Report” (22) included the need to apply integrated, sustainable solutions related to population, agriculture and food security, biodiversity, energy choices, and more. Principles and assessment of sustainability, food processing applications, food manufacturing operations, and food distribution and consumption have been discussed (23).

Gustafson et al. (24) introduced the seven indicators that relate to sustainability. These were adapted by Chen et al. (25), who evaluated sustainability against seven domains (nutrition, environment, food affordability and availability, sociocultural well-being, resilience, food safety, and waste). Sustainability has also been examined through the lens of product and nutrient demands, while incorporating interlinkages between different food supply chains, considering sourcing, processing and transportation, environmental aspects (e.g., land use, climate change, fossil fuel depletion, etc.), and costs, with underpinning Life Cycle Assessment (26).

The use of different indicators and methodology may lead to different conclusions and priorities for action. Therefore, there needs to be consensus among various stakeholders on the choice of the sustainability indicators and performance targets. International standardization for global food supply chains is important for monitoring performance against sustainability targets, regulatory compliance, and consumer communication (27). Recently, a sourcing strategy along the food chain, reflecting nutritional and sustainable aspects and leading to an ingredient branding concept, has been proposed (28). Chen et al. (25) demonstrated the relationship between dietary guidelines and food sustainability.

## Sustainable Food Sources

There is a need for the development of renewable and sustainable sources of food. A significant challenge is to meet the increasing global demand for proteins sustainably. Traditional sources of protein are of vegetal origin (57%) or from meat (18%), dairy (10%), fish and shellfish (6%), and other animal products (9%) (29). The total global consumption of animal proteins is expected to be increased by ~70% from 2007 to 2030 (30). There is increasing demand also for plant-based proteins due to the negative consumer perception of animal sources of protein, increased consumer demand for vegetarian options, and an aversion in Western cultures about insect-based protein sources for food applications (31). Animal-based foods produce higher levels of greenhouse gases (GHGs) than plant-based foods. Greenhouse gases are also associated with climate change (32). The increased demand for animal-based protein is expected to intensify pressure on land due to the need to produce more animal feed (33). This in turn will increase the conversion of forests, wetlands, and natural grasslands into agricultural lands, which has negative consequences for GHG emissions, biodiversity, pollution, and other ecosystem health indicators. Climate change effects on agriculture are expected to threaten the global production of plant protein products from traditional sources (i.e., cereals, legumes).

Other novel sources of protein include alternative plants, aquatic photosynthetic organisms, microorganisms, and insects. There is interest in the cactus pear as it requires fewer inputs (water, nutrients) than traditional sources (cereals, legumes), offers the opportunity for valorization of biomass commonly treated as waste (cladodes), and has the potential for cultivation in arid and semiarid areas (34). Aquatic photosynthetic organisms (microalgae, cyanobacteria, duckweed) do not directly compete with food crops for land and water and have the advantages of year-round harvesting capability, high biomass yields, ability to be cultivated on non-arable land utilizing non-irrigation water (brackish water or seawater), higher protein yield, and resistance to pest and diseases (35). Microorganisms can increase the protein content of organic substrates, offering the opportunity to valorize biomass currently treated as waste (e.g., cladodes, seafood residues, and wastes). Insects do not compete for land, require less water and emit lower GHG and NH_3_, have higher percentage of body weight (up to 80% protein), and are edible and are more digestible than regular livestock (36). Insects perform better in terms of feed conversion efficiency, and they reproduce rapidly, and they can mitigate risk of transmitting zoonotic diseases to humans (37).

Approximately a third of the food produced is wasted (38). An emerging source of new ingredients for the food processing industry is the edible portion of food; this is currently wasted to the food supply. Steps should be taken to recover and reuse edible biomass that is currently wasted to make it a new source of raw materials for food processing (39). Reducing food loss and waste should be part of the solution for sustainable food systems, alongside other strategies to increase agricultural productivity (16). An assessment of environmental as well as economic costs for implementing interventions to reduce food waste is necessary to guide the prioritization of beneficial interventions (40).

## Sustainable Food Processing

Historical developments in food processing have been centered on thermal processing and utilization of natural renewable resources. These include the use of low or high temperature for processing and preservation, with William Cullen introducing the first artificial cold process in the 1750s to present-day energy-efficient refrigeration and freezing systems. Similarly, in early 1800s was the first use of heat for food preservation by Nicholas Appert in response to Napoleon Bonaparte requirements to feed the French army, which has now progressed to modern energy-efficient thermal-processing equipment.

**Figure 1:**
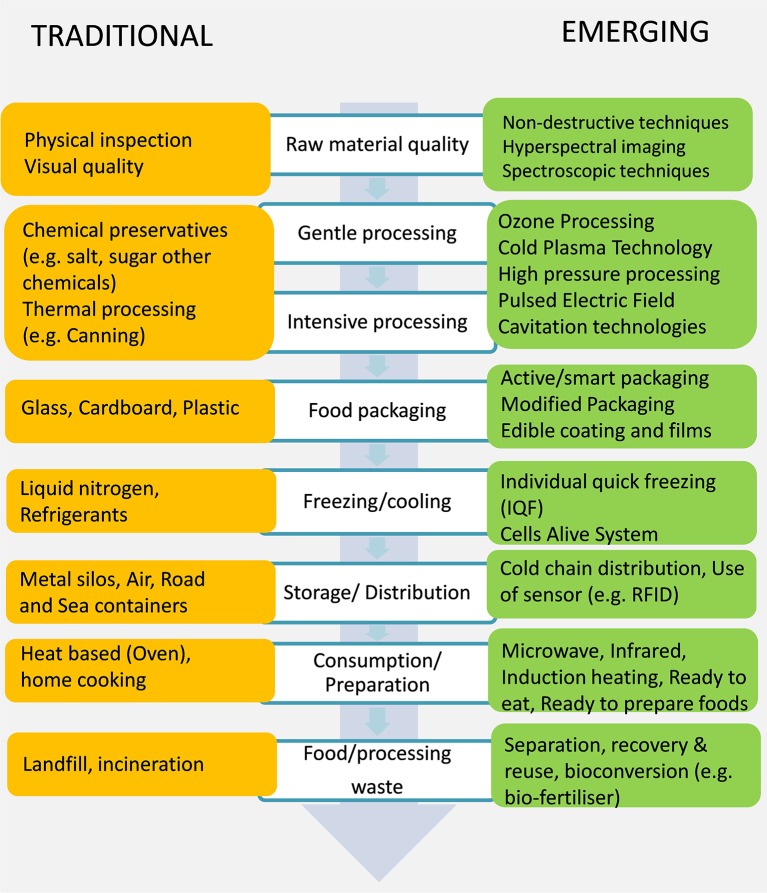
Traditional and emerging technologies and approaches used along the food chain.

Figure 1 provides a summary of major conventional and emerging processes that can be used for developing products in food processing and supply chain. The key existing and potential sources for food process operations are summarized in Table 1. Emerging processes, such as high-pressure processing, pulsed electric field (PEF), pulsed lights, cold atmospheric plasma, microwave, ohmic heating, and ultrasound, are being pursued as alternatives as they are seen as more sustainable processes. The main motivation for the development of emerging, gentle, and mainly non-thermal technologies was to find alternatives or synergies with traditional thermal and chemical preservation processes (41–43). There is also high-pressure assisted sterilization (44), as well as PEF-supported sterilization processes (45). Toepfl et al. (46) showed the potential of high hydrostatic pressure and PEFs for energy-efficient and environmentally friendly food processing including the improvement of cell membrane disintegration and of mass transfer by PEFs, such as improved drying rates for foods or minimizing excess sludge production during waste water treatment, as well as alternative PEF-assisted process developments for the energy intensive beet sugar processing. Green processing food processing techniques, with the main emphasis on extraction processes, were presented by Chemat et al. (47). Henchion et al. (48) provided a review of strategies and factors impacting the sustainability of future protein supply. Lillford and Hermansson (17) identified the design of sustainable process and system engineering influencing novel processes using reduced water and energy and elimination of waste in production, distribution, and consumption as two of their seven key missions regarding global challenges and critical needs of food science and technology.

Table 1Existing natural elements used **(A)** and potential sources for **(B)** food processing.

**Table:** 

**(A)**				
**Sun**	**Air/wind**	**Light**	**Water**	**Earth**
Heat	Flow	Electric energy	Wave power/tidal	Pressure
Light	Gases	Heat	Flow	Temperature/geothermal
Wavelengths	Dense gases	Fire	Hydroelectric	Gravity
Radiation	(Ultra) sound	Plasma	Ice and modifications	Sand and soil organisms
Conductivity	Pressure	Ozone	Supercritical water	Biopolymers
	Vacuum		Hydrostatic pressure	Minerals
			Temperature	Acids
			Conductivity	Lye
			Organisms	
			Biopolymers	
			Acids

**Table:** 

**(B)**		
**New raw materials**	**New processes**	**New packaging materials**
Underexploited and ancient plants	Combination processes	Marine biopolymers
Insects	Diverse pulsed energy processes	Microbial platform chemicals
Leaves	Gravity and magnetism (low/high)	Renewable sources
Aquatic and marine organisms	Wavelengths (all)	
Artic/Antarctic organisms	Gasses (all)	
Cell cultures	Robust, scalable, and flexible processes	
Root cultures	Appropriate/intermediate technologies	
Microbial biomass	Food structuring for property generation	
Edible food losses and waste	Consumer-driven technologies	

Table 1 lists potential sources for food processing that may be considered for improving the sustainability of the food supply. Sustainability, as a given and integral part of food processing, as well as the development of zero discard/loss/waste technologies, needs to be the goal of future directions in the field. Examples for future directions include the exploitation of liquids under negative pressures (49), pressure freezing–air drying (50), high hydrostatic pressure–assisted freezing and thawing (51), re-evaluation of strong magnetic fields (52), or expansion of pulsed process application beyond pulsed light and electric fields (53). There is a need to test the effectiveness of other gas combinations besides the current sources (mainly argon and nitrogen) that take advantage of readily available energy sources, such as air and solar energy, including the development of intelligent combination processes, such as in solar dehydration. In addition, the use of modern, highly effective solar collectors for process equipment improvements, such as solar-driven extruders, water decontamination units or refrigerators, and improved bioconversion technologies to battle toxins (e.g., aflatoxins), is needed.

The application of nanotechnology in food has potential to contribute to sustainable food chains. Nanotechnology encompasses understanding and control of matter on the nanometer scale, which enables alteration of the material properties of products to suit purpose and improve functionality. Nanotechnological innovations in the food and agriculture include encapsulated ingredients that provide protection of sensitive bioactives (e.g., omega-3 fatty acids, vitamins) and increased nutrient delivery, nanomaterials for controlled delivery of anti-microbials, smart sensors for improved food safety management, and nanocomposites for improving barrier properties of packaging materials (54–56). Innovations in delivery systems have a positive impact on sustainability by efficient use of nutrients and functional components/actives through improved ingredient/product performance in the intended application. Smart sensors help inform timely decision making for appropriate interventions, and superior packaging materials can potentially reduce wastage in the supply chain. An assessment of the safety of nanostructured materials coupled with appropriate regulation and legislation will be necessary to facilitate uptake of nanotechnological advances and consumer acceptance (57).

The exploitation of by-products of insect protein production including “gut” enzymes and microbiota seems worthwhile exploring, as well as the plant-soil microorganisms' systems, plant root cultures for secondary metabolite generation, or processing-related improvements of probiotics and prebiotics (58). Finally, a better understanding of existing phenomena as exemplified by France's “The plant as inventor” (59) can serve as a helpful guide for future food process research and development.

## Challenges for Sustainable Food Value Chains

The integration of sustainability should be an additional and new dimension of food security (60). A multidisciplinary approach is required to understand consumption patterns, social norms, behavior, and lifestyles. This approach will help develop acceptable transitions to reduce waste and lower ecological footprints and carbon emissions to develop food ecosystems for sustainable urbanization (61).

- There are challenges for ensuring sustainable food systems for the increasing urban population and transformation in rural–urban linkages (62). This requires integration of rural sectors and better connectivity to the cities, while creating opportunities for economic development, employment, and rural food security. The science-based development of urban ecological systems needs attention especially for climate change adaptation (63).
- Another challenge is to obtain consensus on sustainability indicators for food processors. There is wide variation in reporting by food processors (64). There should be mandatory reporting on agreed indicators, followed by corrective action when appropriate. This includes the need to address Life Cycle Analyses of food processes and processed foods as exemplified by Smetana et al. (65). The issues of transport of raw materials and food also need increased attention (66).
- There must be greater attention paid to the development of business models using an interdisciplinary approach to develop convincing business cases for multiple stakeholders (67). While there has been agreement about the need for adoption of strategies for improving closed loop systems for a circular bio economy, uptake, and realization of the opportunities have been slow.
- An evidence-based nutrition and health-related contribution of processed food are urgently needed (68). There are attempts to initiate such interdisciplinary discussions and activities (69, 70). Greater interaction and communication between the fields of food science and nutrition science are necessary (4, 17).
- Integration of the entire food value chain, which will require concerted interdisciplinary activities (71), is a significant challenge, especially as we move to develop a sustainable and responsible food chain from “precision” agriculture to “precision” food waste and water management (72).
- Future resource-efficient food processing will also need to concentrate and take advantage of the existing biosystems from microorganism–host (plant, animal, human) as well as food–microbiome–human interactions (73). This needs to also include edible microbial biomass (74), as well as enzymes and culture techniques (68) or the use of fermentation processes to reduce toxins (75).

## Conclusions and Recommendations

Significant challenges exist for ensuring sustainability across food chains given the complex nature and variability in global food systems. A key requirement is the establishment of harmonized sustainability indicators that objectivity provide relevant measures for sustainable systems. The design of sustainable processes and engineering systems should embrace the use of emerging processes, which reduces water and energy use.

We recommend the following strategies for improving the sustainability of food systems:

- Resource management—manage food and water scarcity; reduce waste, retain, and recover/reuse nutrients within the food chain; generate a worldwide compendium of indigenous and traditional raw materials, processing, and preparation methods.
- Sustainable processing and improved food delivery—develop sustainable, efficient, and responsible food packaging, storage, transportation, and delivery systems; exploit alternative energy sources and biosystem-based production/processing; build sustainable practices into food preparation and processing; create flexible, scalable, and appropriate urban food processing, preparation, delivery, and consumption models; develop food processes based on PAN (preferences, acceptance, and nutritional needs) principles for consumers (http://etp.ciaa.eu).
- Influencing behavior and developing consumer trust—encourage sustainable and responsible processing, preparation, and consumption of foods; improve transparency and gain consumer trust by providing consumers unbiased information.
- Integration along the food value chain—reevaluate existing food chains and improve integration along the food supply chain to improve sustainability; create a systems approach for the agricultural food chain; promote digital transformation and development of a “precision” food chain; expand interdisciplinary and intradisciplinary food research and development; and involve multiple stakeholders from agriculture, nutrition, trade, government, and consumer organizations.

Creating safe operating spaces for exploited natural systems has highlighted the importance of the interaction of knowledge infrastructure (beliefs, perceptions, models, data), practical processes, and ecological dynamics for informing policy at various scales (76). It might be expected that a similar approach may be considered for defining the safe operating spaces for sustainable food processing that minimize use of resources, consider recycling of inputs for processing (e.g., water, energy), and reduces waste. Implementing transformative sustainability solutions based on advances in science and technology also requires consideration of social and cultural acceptance of changes proposed (77), gaining consumer trust and the development of an appropriate organizational governance framework for prioritizing sustainability initiatives, which takes into consideration the water—energy—food nexus (78, 79).

## Author Contributions

All authors listed have made a substantial, direct and intellectual contribution to the work, and approved it for publication.

### Conflict of Interest

The authors declare that the research was conducted in the absence of any commercial or financial relationships that could be construed as a potential conflict of interest.
